# Immunopathogenic Background of Pars Planitis

**DOI:** 10.1007/s00005-015-0361-y

**Published:** 2015-10-05

**Authors:** Joanna Przeździecka-Dołyk, Agnieszka Węgrzyn, Anna Turno-Kręcicka, Marta Misiuk-Hojło

**Affiliations:** Department and Clinic of Ophthalmology, Wroclaw Medical University, Borowska 213, 50-556 Wrocław, Poland; Department of Internal Medicine, Jagiellonian University, Kraków, Poland

**Keywords:** Pars planitis, Idiopathic uveitis intermedialis, Iris, Ciliary body, Immunology, Eye

## Abstract

Pars planitis is defined as an intermediate uveitis of unknown background of systemic disease with characteristic formations such as vitreous snowballs, snowbanks and changes in peripheral retina. The incidence of pars planitis varies 2.4–15.4 % of the uveitis patients. The pathogenesis of the disease is to be determined in future. Clinical and histopathological findings suggest an autoimmune etiology, most likely as a reaction to endogenous antigen of unknown source, with T cells predominant in both vitreous and pars plana infiltrations. T cells subsets play an important role as a memory-effector peripheral cell. Snowbanks are formed as an effect of post inflammatory glial proliferation of fibrous astrocytes. There is also a genetic predisposition for pars planitis by human leukocyte antigen and several other genes. A coexistence of multiple sclerosis and optic neuritis has been described in numerous studies. Epiretinal membrane, cataract, cystoid macular edema, retinal detachment, retinal vasculitis, neovascularization, vitreous peripheral traction, peripheral hole formation, vitreous hemorrhage, disc edema are common complications observed in pars planitis. There is a need to expand the knowledge of the pathogenic and immunologic background of the pars planitis to create an accurate pharmacological treatment.

## Introduction

Intermediate uveitis described by the Standardization of Uveitis Nomenclature is the inflammation in the anterior vitreous, ciliary body and the peripheral retina, the vitreous is the major site of the inflammation. If there is a systemic disease (e.g., sarcoidosis) or associated infection (e.g., Lyme disease) (Bloch-Michel and Nussenblatt 1987; Jabs et al. 2005), the term idiopathic should not be used. There are many names that have been used to describe inflammation in the anterior vitreous, ciliary body and peripheral retina such as intermediate uveitis, pars planitis, chronic cyclitis, peripheral uveitis, vitritis, cyclochorioretinitis, chronic posterior cyclitis and peripheral uveoretinitis (Foster and Vitale 2013).

The diagnostic term “pars planitis” should be used only for an intermediate uveitis of unknown systemic disease (absence of any known systemic cause) or not related to any infection and where the snowbank or snowball formation is observed (Jabs et al. 2005).

There are limited data on incidence of pars planitis itself. Usually the reported epidemiology underlines incidence of intermediate uveitis (varying between 1.4 and 22 %) as a wider group and not pars planitis (Chan et al. 2007; Foster and Vitale 2013; Henderly et al. 1987; McCannel et al. 1996; Rodriguez et al. 1996).

The pathogenesis of the disease is to be determined in future. Clinical and histopathological findings suggest an autoimmune etiology, most likely as a reaction to endogenous antigen of unknown source, with T cells predominant in both vitreous and pars plana infiltrations. There is limited number of research where the study group includes only patients with pars planitis. Usually research includes patients with intermediate uveitis to the study group and sometimes subtracts a subgroup of patients with pars planitis.

The course of pars planitis and possible complications are well defined and were observed in many trials (Babu and Rathinam 2010; Donaldson et al. 2007; Eichenbaum et al. 1988; Felder and Brockhurst 1982; Foster and Vitale 2013; Green et al. 1981; Jabs et al. 2005; Kenyon et al. 1975; Malinowski et al. 1993a; Nussenblatt and Palestine 1989; Pederson et al. 1978; Pruett et al. 1974; Smith et al. 1976). However, the pathophysiology and possible impact of immune system to the course of the disease and to the complication are to be determined.

Aim of this paper is to summarize available knowledge of immunopathogenesis of pars planitis. The worldwide resources were searched using Medline, Web of Science, Scopus and worldwide websites. Searched terms include: “pars planitis”, “intermediate uveitis”, “uveitis” combined with “immunology”, “immune response”, “T cell”, “B cell”, “innate lymphoid cell” or with “flow cytometry” and in additional search only “experimental models of uveitis” as a single term searched.

Numerous papers were found but only few matched both criteria (approximately 25 items were founded). During the search for “experimental models of uveitis”, task was easier to accomplish and items that were founded were numerous; approximately 17 of them were accurate for probable experimental models of intermediate uveitis or pars planitis. Founded papers focused on different aspects of the disease and define the study group differently. Most of them as patients with intermediate uveitis, some are subtracting a subgroup including patients suffering from pars planitis and few define study group as patients with pars planitis in the strict sense of this term presented previously.

Obtained data will be presented in sections entitled: epidemiology, pathogenesis, clinical features, complications and summary.

## Epidemiology

Intermediate uveitis has been reported in 1.4–22 % of uveitis patients and there are limited data on the incidence of pars planitis (Chan et al. 2007; Foster and Vitale 2013; Henderly et al. 1987; McCannel et al. 1996; Rodriguez et al. 1996). The rate of 1.5 per 100,000 per year was reported by The Northern California Epidemiology of Uveitis Study (Gritz and Wong 2004). In tertiary referral centers, 2.4–15.4 % of uveitis patients have been reported as the one with pars planitis diagnosis, but it must be taken under consideration that these centers observed more severe end of the spectrum of ocular inflammatory disease (Henderly et al. 1987; Wakefield et al. 1986).

## Pathogenesis

### Immunological Background

In healthy eye, an outer blood–retinal barrier (formed by tight junction of retinal pigmented epithelial cells and fenestrated choriocapillaris) as well as a blood-aqueous barrier (formed by tight junction of non-pigmented ciliary body epithelium and fenestrated endothelial cells) are considered responsible for immunological privilege. The inner blood–retinal barrier (formed by tight junction, non-fenestrated endothelium and astrocyte and Muller cell foot branches) acts as a true barrier (Shechter et al. 2013). Numerous immunomodulatory mediators, including: transforming growth factor (TGF)-β, membrane-bound TGF-β, interleukin (IL)-10, CD86, somatostatin, the enzyme thrombospondin, the apoptotic mediators FAS antigen ligand, tumor necrosis factor (TNF)-related apoptosis-inducting ligand, glucocorticoid-induced TNF-receptor-related protein, programmed cell death 1 ligand, non-classical MHC class Ib molecules and the enzyme indoleamine-2,3-dioxygenase can be found in both the outer blood–retinal barrier and the blood-aqueous barrier epithelial milieu (Kaur et al. 2008; Kerr et al. 2008; Streilein 2003; Sugita 2009; Sugita et al. 2006a; Sugita and Streilein 2003). Additionally, the iris, ciliary body and retinal pigment epithelium cells showed in vitro an ability to suppress the proliferation and activation of T cells either by direct cell to cell contact or by soluble factor-mediated mechanisms (Gregerson et al. 2007; Ishida et al. 2003; Kaur et al. 2008; Kerr et al. 2008; Sugita 2009; Sugita et al. 2006a). In some cases, retinal pigment epithelium (RPE) cells might convert effector T helper (Th)1 cells into regulatory T (Treg) cells (Sugita et al. 2004, 2006b; Yoshida et al. 2000a, b). RPE can also down regulate Th1 (Sugita et al. 2004), Th17 (Sugita et al. 2011), Th22 (Sugita et al. 2013b), CD8^+^ T cells (Sugita et al. 2006c), B cells (Sugita et al. 2010), macrophages (Zamiri et al. 2006) and dendritic cell (Sugita et al. 2013a) as well as up regulate Treg (Sugita et al. 2009, 2008) and suppressor myeloid cells (Tu et al. 2012).

Histopathological and clinical findings suggest a possible autoimmune etiology of pars planitis. In the infiltrate surrounding of the retinal vessels and in the snowbanks, the Th cells have been found. They can be possibly directed against some unknown ocular antigens (Wetzig et al. 1988; Yoser et al. 1992). The vitreoretinal interface, associated with retinal blood vessels, ciliary body, or the pars plana itself, may be the source of sought antigens (Davis et al. 1992).

Several experimental models of uveitis were described, used especially in cases of posterior uveitis, induced by a subcutaneous injection of single dose of soluble antigens (usually retinal). Mentioned models (see Table 1) include: experimental autoimmune uveitis (EAU) (Adamus and Chan 2002; Chan et al. 1985a, b; de Kozak et al. 1981), experimental melanin protein-induced uveitis (EMIU) (Adamus and Chan 2002; Matteson et al. 1999), experimental autoimmune encephalitis associated to anterior uveitis (EAE/AU) (Adamus et al. 2000; Adamus and Chan 2002; Buenafe et al. 1998) and endotoxin-induced uveitis (EIU) (Li et al. 1995). In each model, different soluble antigen is used; S-arrestin or interphotoreceptor retinoid-binding protein (IRBP), melanin proteins, myelin basic protein and lipopolysaccharide, respectively, for EAU, EMIU, EAE/AU and EIU (Gasparin et al. 2012). None of the described models fully explains the changes that occur in humans, especially in case of pars planitis. The possible candidates for pars planitis antigens were specified in conducted experiments involving humans. Those include human lens protein fraction (Doycheva et al. 2007), HNK-1 carbohydrate epitope (Uusitalo et al. 2000) and beta B1-crystallin (Stempel et al. 2003).

**Table 1:** Experimental models of uveitis—summary

Experimental model	Antigen	Target	Immunological response		References
			Cellular	Humoral	
Experimental autoimmune uveitis	S-antigen/S-arrestin/S–Ag Interphotoreceptor retinoid-binding protein/IRBP Rhodopsin and its illuminated form, opsin Recoverin Phosducin	Photoreceptors	T cell-mediated inflammatory response present, mainly CD4^+^ cells Early stages Th/inducer lymphocytes vs T-suppressor/cytotoxic cells ratio is as low as 5 to 1 Later stages the ratio changes to 1/1, or even ½	IL-2, IFN-γ and TNF-α are produced by the subset Th1	Adamus and Chan (2002); Chan et al. (1985a, b); de Kozak et al. (1981)
Experimental melanin protein-induced uveitis	Melanin protein	Uveal melanocytes	CD4^+^ T cells and macrophages—the primary mediators of the inflammatory response	Early stages—IL-2 and IFN-γ Medium stage—IL-12 The peak of the inflammation—TNF-α Other inflammatory mediators such as nitric oxide can be also involved	Adamus and Chan (2002); Matteson et al. (1999)
Experimental autoimmune encephalitis associated to anterior uveitis	Mielin binding protein	Iris myelinated nerves and spinal cord	Th1 cells Clinically—uveitis has longer duration and uveitis is still ongoing even after the encephalitis resolves	IL-2 and IFN-γ are produced by the subset Th1	Adamus et al. (2000); Adamus and Chan (2002); Buenafe et al. (1998)
Endotoxin-induced uveitis	Lipopolysaccharide (LPS)		Intense acute inflammatory cellular infiltration of neutrophils and macrophages Peak at 18–24 h after LPS injection and lasts for 72 h T cells also play a role in EIU	Released cytokines: TNF-α, IFN-γ, TGF-β, IL-1, IL-6, IL-8, CCL2 and CCL5	Adamus and Chan (2002); Li et al. (1995)

The exact cause of pars planitis remains unknown (see Table 2), but CD4^+^ T cells that express an activation marker CD69 (an early activation marker) in either peripheral blood (Murphy et al. 2004) or in the aqueous humor are found, respectively, in patients with pars planitis and with idiopathic uveitis (Calder et al. 1999). Longitudinal measurements over a period of treatment rather than single evaluation of CD4^+^CD69^+^ T cells may be a useful substitute marker of ocular inflammation. As such it may be used to asses disease activity, but further studies are needed (Kilmartin et al. 2001; Murphy et al. 2004).

**Table 2:** Immunopathogenesis of pars planitis/idiopathic intermediate uveitis—summary (included manuscripts in which the study group or its subdivided subgroup in the description given by the authors can be considered as pars planitis)

References	Number of patients	Findings	Materials	Method
Murphy et al. (2004)	Idiopathic intermediate uveitis = 18 Sarcoid intermediate uveitis = 6 Control group = 23	The expression of the CD69 and TNF-α by peripheral blood CD4^+^ lymphocytes of patients with idiopathic intermediate uveitis was significantly higher than control subjects (*p* = 0.002) No differences were found in the other variables measured between the idiopathic intermediate uveitis and normal controls (even when the group subdivided into active and inactive subgroups)	PB lymphocytes; 14 of the patients were on systemic immunosuppressive therapy, 4 were on topical steroids and 6 were without treatment^a^	CD4^+^ T cell expression of CD69, CCR4, CCR5, CXCR3 and the intracellular cytokines IFN-γ, TNF-α, and IL-10 by flow cytometry IL-2, IL-4, IL-5, IL-10, IFN-γ, and TNF-α production following unstimulated and activated culture using cytokine bead array
Klok et al. (1998)	Idiopathic intermediate uveitis = 61 Uveitis control = 143 Control group = 29	Increased serum IL-8 was found in patients with idiopathic intermediate uveitis (*p* < 0.01) compared to control group. Raised IL-8 levels in idiopathic intermediate uveitis were significantly associated with active disease (*p* < 0.001) and the presence of vitreous exudates (*p* < 0.001), papillitis, and periphlebitis (*p* < 0.01) compared to control group	PB samples	Serum levels of CRP were assessed using an immunodiffusion assay and the IL-8 was measured with ELISA
Klok et al. (1999)	Idiopathic intermediate uveitis = 61 Uveitis patients with systemic disease = 56 Uveitis patients without systemic disease = 58 Control group = 21	Increased serum levels of sICAM-1 were found in idiopathic intermediate uveitis compared with toxoplasmosis, Fuchs’ cyclitis, and healthy controls (*p* < 0.001). Raised sICAM-1 levels in the idiopathic intermediate uveitis group were associated with active ocular disease (*p* < 0.01) and the presence of vitreous exudates (*p* < 0.05)	PB samples	Serum levels of sICAM-1 were detected using a commercially available ELISA
Deschênes et al. (1988)	Uveitis = 51 (AqH or VB or/and PB) Control group PB = 14 Uveitis group contain 8 patient with pars planitis (PB = 8, AqH = 1)	The diagnosis of pars planitis was indicated in the study but no statistical analysis was made for that subgroup Uveitis group had more activated T cells (IL-2R^+^) in the PB than control (*p* < 0.05). The diagnosis of idiopathic uveitis correlated with increased expression of the IL-2 receptors on peripheral Leu-3A^+^ lymphocytes (correlation = 0.247, *p* = 0.033) and with increased IL2^+^Leu2A lymphocytes in AqH (a negative correlation = −0.572, *p* = 0.049)	AqH, VB and PB samples	Two colors cytometric analyses of cell population were performed
Pedroza-Seres et al. (2007)	Pars planitis = 15 Control group = 15	CD57^+^ T cells subsets were increased in patients with pars planitis (*p* = 0.002). A greater number of CD8^+^CD57^+^ T cells than CD8^+^CD57^−^ T cells were positive to perforin (*p* = 0.006) and granzyme-A (*p* = 0.01) in pars planitis group than in control	PB lymphocytes	The CD57^+^ T cell subsets CCR7, CD27, CD28, CD45RA, CD45RO, intracellular IFN-γ, IL-4, perforin and granzyme-A expression were assessed by flow cytometry

*AqH* aqueous humor, *PB* peripheral blood, *VB* vitreal body, *sICAM-1* soluble intracellular adhesion molecule 1

^**a**^Researchers do not specify uveitic groups

There are some studies showing increase in levels of surrogate markers of immune activation in the serum of patients with intermediate uveitis. These markers include IL-8 and soluble intracellular adhesion molecule 1 (Klok et al. 1998, 1999) as well as increased expression on peripheral CD4^+^ T cells such as soluble IL-2 receptor, HLA-DR and CD25 of patients with uveitis (Deschênes et al. 1988; Dick et al. 1992; Feron et al. 1995). All of mentioned above need to be confirmed in longitudinal studies before they become useful in clinical practice, in predicting an outcome of the disease and monitoring of the immunosuppressive treatment.

Studies of the model of experimental autoimmune uveitis have shown that Th1-related cytokines predominate in the active phase of the disease and IL-10 during resolution phase, indicating a shift towards Th2 (Barton et al. 1995). Further studies of peripheral blood CD4^+^ T cells expression of chemokines show no difference between the Th1-associated chemokine receptors CXCR3 and CCR5 or the Th2-associated chemokine receptor CCR4 in patients suffering from intermediate uveitis (enrollment of patients includes idiopathic and presumed sarcoid intermediate uveitis) compared to healthy controls as well as active compared to inactive disease (Murphy et al. 2004). Murphy et al. (2004) described an increase in the intracellular CD4^+^ T cell expression of TNF-α in idiopathic intermediate uveitis which suggests a predominant CD4^+^ T cell subset and a new potential role of anti-TNF-α treatment in this type of uveitis (Sugita et al. 2006a).

In animal models of experimental autoimmune uveoretinitis, a transfer of CD4^+^CD25^high^Foxp3^+^ Treg cells confer protection from uveitis induced by the uveitogenic retinal antigen IRBP (Keino et al. 2007; Siepmann et al. 2007; Silver et al. 2007). The lower percentage of CD4^+^Foxp3^+^ T cells was observed in patients with active uveitis compared to inactive. However, no statistically significant differences were found in percentage of CD4^+^Foxp3^+^ T cells between patients with known systemic autoimmune disease compared to idiopathic uveitis. The outcome was this same for patients with cystoid macular edema compared with no evidence of cystoid macular edema. In addition, no linear correlation of the percentage of CD4^+^Foxp3^+^ T cells with logMAR best corrected visual acuity was observed (Yeh et al. 2009).

Until now, little is known about the role of CD8^+^ T cells in pars planitis, and of coexpression of functional NK receptors, like CD56 or CD57 on CD8^+^ T cells, which are thought to be associated with effector function of these cells (Ahn et al. 2005; Tarazona et al. 2000). Particularly CD57 expressed on both CD4^+^ and CD8^+^ T cells is considered to be correlated with late immune responses (d’Angeac et al. 1994). Many studies have described an association between CD57 expression and persistent antigenic stimulation and chronic diseases. Pedroza-Seres et al. (2007) described in their paper a subset of CD57^+^ T cells. CD57^+^ T cells had a peripheral memory phenotype (CCR7^−^CD27^−^CD28^−^). The role of these cells in helper cell regulation was evaluated by cytokine production in CD57^+^ T cells. The intracellular proteins involved in cytotoxicity were measured in CD8^+^CD57^+^ T cells and its elevated expression may suggest that these cells play an effector role in pars planitis (Pedroza-Seres et al. 2007). Also, in the model of experimental autoimmune uveitis, CD8^+^ T cells can be stimulated by IRPB and TGF-β1 to express Foxp3 in high levels and may have suppressive functions (Peng et al. 2007).

Role of B cells was evaluated in studies performed on enucleated eyes, in case of infiltration of these cells in pars plana, vitreous and retina, but the results were not compared to immunological findings in the peripheral blood. In addition, the enucleated eyes were in last stage of the disease and nowadays with more efficient immunosuppressive treatment and wider range of possible surgical options, the stages usually are not recorded. Finally, the researches were conducted in the eighties of twentieth century and diagnostic possibilities have now improved.

The aspect of innate response in pars planitis was not taken into account in conducted researches, so far. The role of macrophages and neutrophils has not been evaluated so far. In the light of newly described innate lymphoid cells (ILCs) that have a function analogic to Th cells while also including NK cells, there will be a great demand of further research on ILCs in pars planitis.

From the point of view presented above, better understanding of immunological processes taking place in an affected eye during pars planitis may play a key role in pathogenesis of pars planitis and in appropriate treatment of this disease.

### Genetic Background

There is also a genetic predisposition for the pars planitis. Number of studies described the contribution of the human leukocyte antigen (HLA) and several other genes in addition to those of the HLA in the pathogenesis of pars planitis. However, they differ in inclusion and exclusion criteria used to eliminate patients with intermediate uveitis due to other causes (e.g., sarcoidosis, syphilis) and described differences may play an important role why results of studies vary from each other. Several studies claimed the contribution of the HLA alleles considered as susceptibility markers in multiple sclerosis and optic neuritis which are: HLA-DR15, -DR51 and -DQ6 (Hillert et al. 1996; Malinowski et al. 1993b; Oruc et al. 2001; Raja et al. 1999; Tang et al. 1997). The subtype of intermediate uveitis was more often found in patients with multiple sclerosis (Bamford et al. 1978; Giles 1970; Porter 1972). An elevated frequency of HLA-B8, -B51 and -DR-2 was estimated after exclusion of patients with multiple sclerosis from the pars planitis group (Malinowski et al. 1993b). Some authors claimed that HLA-DR15, -DR51 and -DR17 contributed to the etiology of both multiple sclerosis and pars planitis (Oruc et al. 2001; Raja et al. 1999). Some studies evaluated a genetic predisposition of pars planitis and its course, for example, in Mexicans. HLA-A, -B, and -C typing was done on T cells isolated with immunomagnetic beds from a group of untreated pars planitis patients with Mexican origins. The significant increase in frequency was found in DRB1*0802 in these patients. There was an association between clinical features, gender and HLA alleles (shown in Table 3; Alaez et al. 2003). Nonetheless, there can be found some studies that show no correlation between pars planitis and HLA alleles (Greiner et al. 2003; Mantrana-Bermejo et al. 2010).

**Table 3:** Distribution of HLA alleles in pars planitis with association to clinical features (family reports were excluded)

References	Number of patients	Findings	The origin of the patients	Method
Alaez et al. (2003)	Study group = 79 (pars planitis) Control group = 204	Nonsymmetrical onset: 1. Females—HLA-B51 (OR = 9.8) 2 Males—HLA-B60 Symmetrical onset: 1. Females—HLA-Cw1 2. Males—HLA-DRB1*0802, HLA-DQA1*0401, HLA-DQB1*0402 Corneal peripheral epitheliopathy: 1. Females—HLA-DRB1*0602 2. Males—correlation possible HLA-DRB1*0602 Totally increased in study group: 1. Females—HLA-B51, HLA-Cw1 2. Males—HLA-DRB1*0802	Mexican	HLA-A, -B, and -C typing on T cells isolated with immunomagnetic beads HLA-DRB1, -DQA1, and -DQB1 loci typing by polymerase chain reaction–sequence-specific oligonucleotide probes
Malinowski et al. (1993b)	Study group = 40 (pars planitis) Control group = 431	In study vs control group HLA-B8 (*p* = 0.011), HLA-B51 (*p* = 0.049), HLA-DR2 (*p* < 0.0001) were found more frequently. HLA-DR2 has been associated with multiple sclerosis**		HLA analysis of class I and II phenotypes
Oruc et al. (2001)	Study group = 28 (pars planitis) Control group = 50	In patients with pars planitis vs control group HLA-DR15 (*p* = 0.0001), HLA-DR51 (*p* = 0.0001) and HLA-DR17 (*p* = 0.0001) were found more frequently		HLA-DR genotyping
Raja et al. (1999)	Study group = 32 (pars planitis) Control group = 1983 (bone marrow donors registered in Johns Hopkins Immunogenetics Laboratory)	HLA-DR15 was associated with pars planitis (*p* = 0.004)	Predominantly white (one Hispanic and one Asian patient)	HLA-DR genotyping
Mantrana-Bermejo et al. (2010)	Study group = 24 (pars planitis) Control group = 194	No association between the occurrence of pars planitis and HLA DR 15 or other known HLA genotypes	Spanish	HLA-A, -B and -DR genotyping
Greiner et al. (2003)	Study group = 15 (pars planitis*) Control group = 34	No association between the occurrence of pars planitis and the HLA DR15 or other known HLA DR genotypes	Scottish	HLA-DR genotyping for all DRB genes using PCR sequence-specific primers

No study group nor control group patients were related in included researches

*OR* odds ratio

* Only patients with bilateral vitritis and snowbanks in at least one eye was included

** An exclusion has been made of five patients with pars planitis in whom multiple sclerosis were developed—this exclusion did not change the significance of above findings

## Clinical Features

Patients with pars planitis usually present with minimal symptoms such as floaters or blurred vision. However, in severe cases, a significant visual loss can occur due to aggregation of floaters in the vitreous or due to macular oedema. The signs of inflammation from the anterior chamber may be minimal in the form of keratic precipitates, flare and cells or they may be absent. Sometimes we can also find posterior synechiae which are usually seen involving the inferior iris. A characteristic feature of pars planitis–vitritis is typically described and evaluated by clinicians as vitreous haze ranging from trace to 4+ (Jabs et al. 2005).

**Fig. 1:**
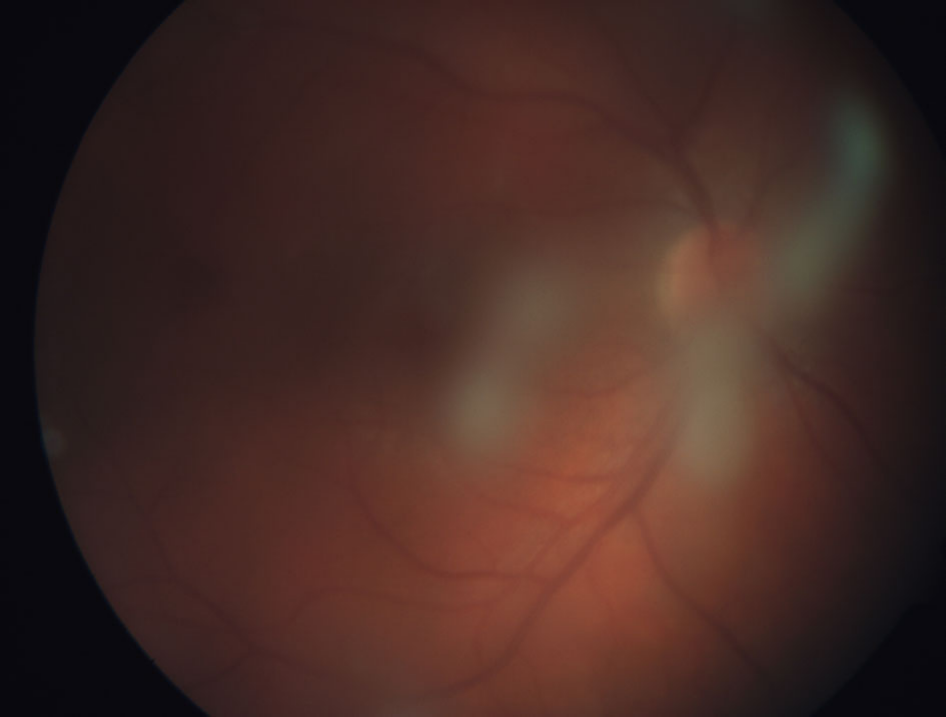
16-year-old feme patient with diagnosed pars planitis, first ophthalmological examination. Snowballs are seen as focal *yellow*–*white* massive exudates in peripheral anterior vitreous

**Fig. 2:**
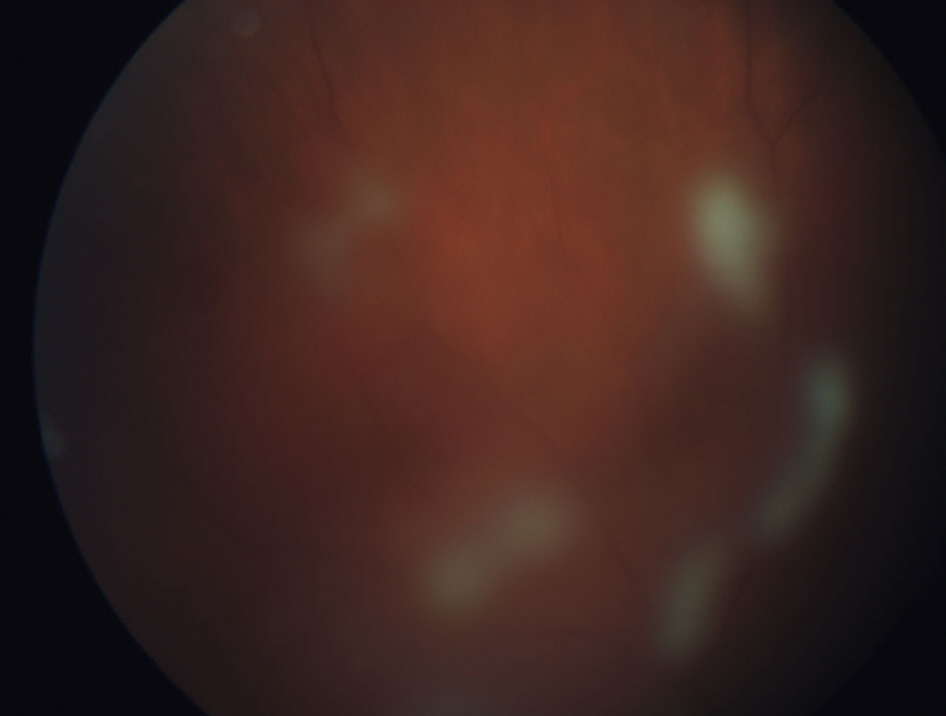
Massive exudates in mid vitreous that block fundoscopic examination of posterior pole

The vitreous snowballs are yellow-white inflammatory aggregates which can be found in the midvitreous and inferior periphery (Felder and Brockhurst 1982; Nussenblatt and Palestine 1989; Pruett et al. 1974) (Figs. 1, 2). Some studies described vitreous snowballs as made of granulomatous inflammation (Green et al. 1981). The difficulty to get the material for further research precisely from intended spot results in lack of many molecular studies.

Snowbanks, the snow-white exudates that are found on the pars plana, usually are seen in the inferior area but they also can extend to 360° of the retinal periphery. Typically they are associated with more severe forms of the disease, and require more aggressive therapy (Felder and Brockhurst 1982; Nussenblatt and Palestine 1989; Pruett et al. 1974). As previously described in histopathologic studies, snowbanks consist mainly of glial elements (probably fibrous astrocytes which can produce a large diameter collagen fibrils and basement membranes) (Eichenbaum et al. 1988; Green et al. 1981; Kenyon et al. 1975; Pederson et al. 1978), it was confirmed by staining with anti-Muller cell antibody and anti-glial fibrillary acid protein (GFAP). The formation of snowbanks is stimulated by inflammation. Activated Muller cells express class II MHC molecules and present antigen to T cells, sustaining an active state of inflammation itself (Roberge et al. 1985). The major collagen glycoproteins of snowbanks are type IV collagen and laminin (formed rather by glial cells than fibroblasts as the letter one’s characteristic products are collagen type I and III). All these facts confirmed a hypothesis of postinflammatory glial proliferation that takes place in eye of pars planitis patients. Different results from the one’s described above can be found in Abu El-Asrar and Geboes (2002) work. In their point of view, the snowbanks are an acellular formation (in exception of uveal side cytokeratin-positive retinal pigment epithelial cells without cells positive for the GFAP and the myofibroblast cell marker alpha smooth muscle actin) containing tenascin and collagen type I, II and III, without the immunoreactivity of laminin or fibronectin (Abu El-Asrar and Geboes 2002).

**Fig. 3:**
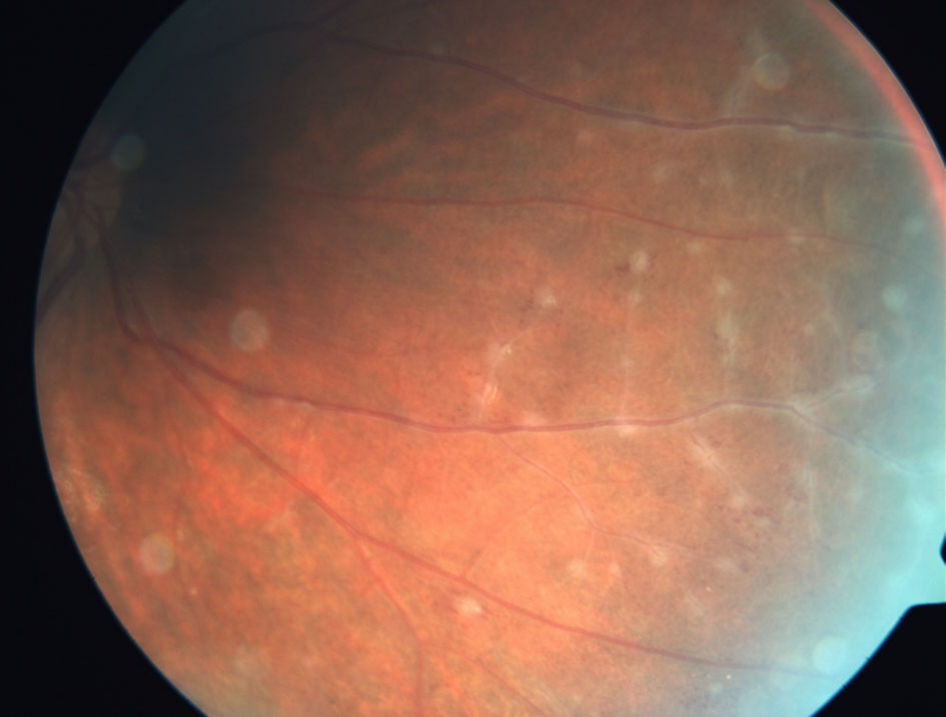
17-year-old female patient with diagnosed multiple sclerosis, first ophthalmological examination. Vascular sheaths also known as vascular cuffs are seen as massive perivascular exudates

Retinal changes in pars planitis occur in its peripheral part and include tortuosity of arterioles and venules, sheathing of veins (Fig. 3), neovascularization and sometimes retinal detachments (Felder and Brockhurst 1982; Nussenblatt and Palestine 1989; Pruett et al. 1974). The vascular sheathing in histopathologic studies presents a cellular infiltration dominated by T cells with few B cells usually posterior to the junction of the iris and ciliary body (Eichenbaum et al. 1988; Wetzig et al. 1988).

## Complications

The most common ocular complication in patients with pars planitis is epiretinal membrane (ERM) with high incidence from 7 to 36 % in different studies. The mean interval between onset of pars planitis and ERM was 6.5–7.9 years (Babu and Rathinam 2010; Donaldson et al. 2007; Malinowski et al. 1993a). The mechanism of epiretinal membrane formation during pars planitis is unknown.

Cataract was also common for pars planitis (frequency 14–30 %), it occured as a result of the disease and the treatment. It was difficult to distinguish between these too situations retrospectively. Most of the diagnosed cataracts were posterior subcapsular cataract followed closely by mixed nuclear sclerotic and posterior subcapsular cataracts. Described complications were developed much later, mean onset after presentation was 9.8 years (Babu and Rathinam 2010; Donaldson et al. 2007; Foster and Vitale 2013; Malinowski et al. 1993a; Pruett et al. 1974; Smith et al. 1976). The mechanism of steroid cataract induction remains unknown, nevertheless the observation of glucocorticoid-induced gene transcription and the finding of a transcriptionally active glucocorticoid receptor in human lens epithelial cells suggest that glucocorticoids can act directly in lens epithelial cells and modulate several signaling pathways influencing gene transcription (Dickerson et al. 1997; Gupta and Wagner 2009; James et al. 2003).

**Fig. 4:**
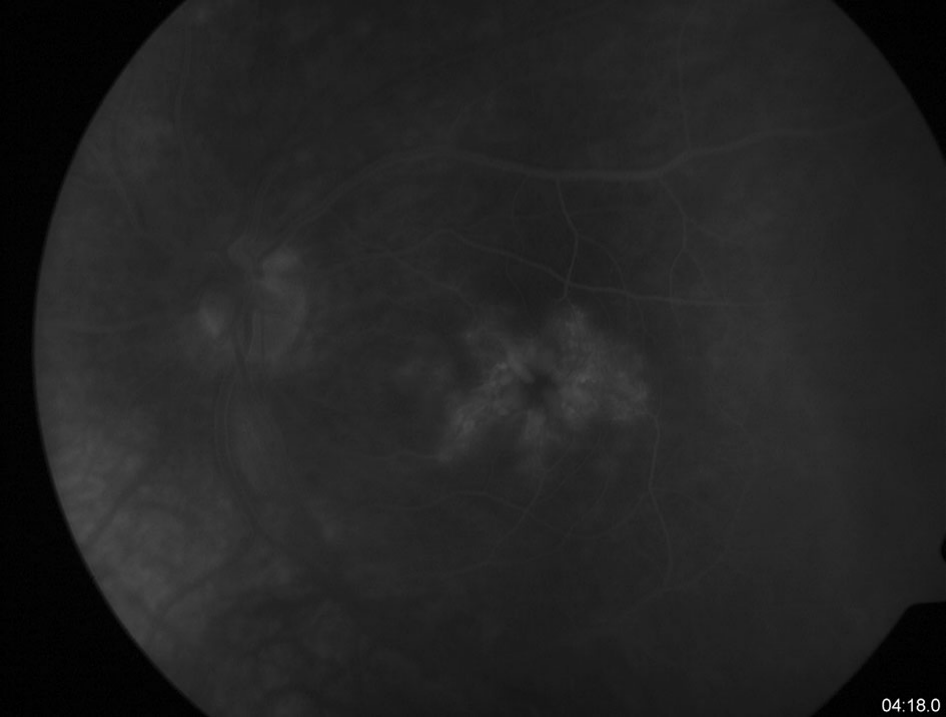
Late phase of fluorescein angiography showing cystoid macular edema in 11-year-old boy with pars planitis

Cystoid macular edema (CME) was described as a major cause of visual loss (incidence 8–26 %) in patients with pars planitis (Fig. 4). The mean interval between onset of pars planitis and CME was 5.7 years (Babu and Rathinam 2010; Donaldson et al. 2007; Foster and Vitale 2013; Malinowski et al. 1993a; Pruett et al. 1974; Smith et al. 1976). The pathophysiology of cystoid macular edema was described in patients with uveitis without subdividing that group (no data show a pathomechanism of CME in patients with pars planitis). Retinal vascular hyperpermeability can be influenced by several mediators such as: nitric oxide, TNF-α, vascular endothelial growth factors, prostaglandins, leukotrienes, cytokines IP-10 and IL-6 (Acharya et al. 2009; Curnow et al. 2005; Markomichelakis et al. 2005; Probst et al. 2004; van Kooij et al. 2006). Vascular leakage could result from endothelial cell damage (Leal et al. 2007).

The isolated cases of other complications occurring during onset of pars planitis as well as intermediate uveitis were registered in large researches, such as: retinal detachment (including exudative retinal detachment), retinal vasculitis, neovascularization, vitreous peripheral traction, peripheral hole formation, vitreous hemorrhage, disc edema, optic neuritis (Brockhurst and Schepens 1968; Deane and Rosenthal 1997; Donaldson et al. 2007; Malinowski et al. 1993a; Prieto et al. 2001; Pruett et al. 1974; Smith et al. 1976).

## Conclusion

Little is known about background and pathology of pars planitis. From the patient and the ophthalmologist point of view, there is a great need for immunological research of this condition to improve the treatment.
